# Supplementary material for: Antibacterial efficacy of cold atmospheric plasma against Enterococcus faecalis planktonic cultures and biofilms in vitro
Source: PLoS One. 2019 Nov 26;14(11):e0223925. doi: 10.1371/journal.pone.0223925 (PMC6879142; doi:10.1371/journal.pone.0223925)
Supplement: S1 File — (DOCX) [file pone.0223925.s001.docx]

**Supporting Information for**

**Antibacterial efficacy of cold atmospheric plasma against *Enterococcus faecalis* planktonic cultures and biofilms *in vitro***

Felix Theinkom , Larissa Singer, Fabian Cieplik, Sylvia Cantzler, Hannes Weilemann, Maximilian Cantzler, Karl-Anton Hiller, Tim Maisch and Julia L. Zimmermann

Data set necessary to replicate study findings for figure 2, figure 3, figure 4, figure 5 and table 1.

**S1 Fig 2.** **Antimicrobial assay towards planktonic *E. faecalis* on agar plates.**

CAP_treatment_period in minutes. CFU_LogRed: Reduction of CFU logarithmic.

N=6 independent experiments

**S2 Fig 3.** **Antimicrobial efficacy of CAP towards *E. faecalis* biofilms cultured for 24 h**

age biofilm in hours; CAP_treatment_period in minutes; CFU_LogRed: Reduction of CFU logarithmic; N=5 independent experiments

**S3 Fig 4.** **Antimicrobial efficacy of positive controls CHX and UV-C radiation towards *E. faecalis* biofilms cultured for 24 h**

 age biofilm in hours; concentration_CHX: [%]; radiation_UVC: dosage in J/cm2; CFU_LogRed: Reduction of CFU logarithmic; N=5 independent experiments.

**S4 Fig 5.** **Spectroscopic measurements for release of nucleic acids upon CAP treatment**

OD_260: OD measurement at 260nm; UC: untreated control, PC: positive control, lysozyme treatment followed by Proteinase K digestion; n= 3 independent experiments.

**S5 Table 1. Results from antimicrobial assay towards *E. faecalis* biofilms cultured for 48 h or 72 h**
