# Supplementary material for: Antibacterial efficacy of cold atmospheric plasma against Enterococcus faecalis planktonic cultures and biofilms in vitro
Source: PLoS One. 2019 Nov 26;14(11):e0223925. doi: 10.1371/journal.pone.0223925 (PMC6879142; doi:10.1371/journal.pone.0223925)
Supplement: Attachment — Submitted filename: Rebuttal letter__2019.docx [file pone.0223925.s002.docx]

| University Hospital Regensburg  Department of Dermatology |  |
| --- | --- |
|  | **Klinik und Poliklinik für DERMATOLOGIE**  Interdisziplinäres Hautkrebszentrum Ostbayern  QM-zertifiziert nach ISO 9001: 2008  **apl. Prof. Dr. rer nat. Tim Maisch**  **antimicrobial CAP and PIB research unit** |

14/08/2019

**Rebuttal Letter to PONE-D-19-18041**

Dear

Monica Cartelle Gestal, PhD
Academic Editor

Please find below our rebuttal letter with a point to point answer to both the academic editor and reviewer’s comments and recommendations.

4. Is the manuscript presented in an intelligible fashion and written in standard English?

PLOS ONE does not copyedit accepted manuscripts, so the language in submitted articles must be clear, correct, and unambiguous. Any typographical or grammatical errors should be corrected at revision, so please note any specific errors here.

Reviewer #1: Yes

Reviewer #2: No

**Answer: We revised our manuscript concerning language.**

**5. Review Comments to the Author**


**Reviewer #1:** Although the idea of using cold plasmas for antimicrobial therapy is not new, the paper is dealing with an important and well investigated subject, antimicrobial effect of such plasmas against one of the ESKAPE pathogens, E. faecalis. This approach could be efficiently used for the therapy of wounds and also in dentistry.
The results are clearly presented and conclusions are supported by experimental data.
I would recommend authors to expand their discussion by adding some details regarding the known effect of cold plasmas against other ESKAPE pathogens, highlighting how would this approach improve the therapy of such infections. Also discussing the antimicrobial mechanisms of cold plasmas against other microbial species, could help in revealing the primary antimicrobial mechanism against *E.faecalis*.

**All changes are marked in yellow within the manuscript** (file labeled as “Revised Manuscript with Track Changes”).

**Answer:**

**We thank the reviewer for the comments. We have inserted some new statements concerning efficacy of CAP against ESKAPE pathogens within the discussion section (page 17, line 365-378). Here in this study we have decided to use *E. faecalis* due to the fact that this pathogen is known to grew and form biofilms on both inanimate and living surfaces and therefore *E. faecalis* gained tolerance and susceptibility against external influences from the environment (page 19, line 426-432).**

**Reviewer #2:** The authors focus to demonstrate possible antimicrobial activity of CAPs on E. faecalis. The authors used CAP in both planktonic and biofilms to investigate the possible antimicrobial effects. The authors reported a decrease in CFUs after 5 and 10 minutes of CAP treatment in 24h and 72h biofilms. The authors suggest that the antimicrobial activity of CAP is not due to damage to cell membranes as they did not observe any DNA release after treatment.

**All changes are marked in green within the manuscript.** (file labeled as “Revised Manuscript with Track Changes”).

My main concerns of the presented manuscript are listed below:
1. The authors failed to explain and elaborate on the importance and possible future application of antimicrobial activity of CAP on E. faecalis. The addition of more elaborate details on the rational of choosing this bacteria to test the antimicrobial activity of CAPs would improve the quality of the introduction.

**Answer:**

**Here in this study we used *E. faecalis* as one the ESKAPE pathogens which is known to be able to grew and form biofilms on both inanimate and living surfaces and therefore *E. faecalis* gained tolerance and susceptibility against external influences from the environment. We have added new statements on page 3, line 61-64.**

2. Although the details of experimental plans are explained thoroughly, there are no explanation of observed results in the result section.

**Answer:**

**We have now revised the results section as recommended.**

3. The statistical analysis are not explained and the tests are not mentioned and the levels of significance are not reported.

**Answer:**

**We thank the reviewer for the comment. Indeed, we did not explain the statistical test and did not mention the level of significance, because we did not perform classic significance analyses. However, we mentioned in the data analysis section our definition of antimicrobial efficacy and disinfection. (L230-234: This passage reads as follows: “Medians on or below these lines mean a bacterial reduction by 3 log_10_ (≥99.9%) or by 5 log_10_ (≥99.999%). According to the guideline of infection control this means a biologically relevant antimicrobial activity or a disinfectant effect, respectively [Boyce JM, Pittet D. Guideline for Hand Hygiene in Health-Care Settings: recommendations of the Healthcare Infection Control Practices Advisory Committee and the HICPAC/SHEA/APIC/IDSA Hand Hygiene Task Force. Infect Control Hosp Epidemiol. 2002;23(12 Suppl):S3-40.]”).**

**From microbiological point of view concerning these definitions of the wordings “antimicrobial” and “disinfection”, we used these definitions as the biological relevant tool to discriminate the data.**

**In addition, thoroughly reviewing our data analysis data section again, we found that the spectroscopic measurements are depicted as median min and max (see fig. 5) and not as medians, first and third quartile. We changed this part accordingly within the data analysis section.**

4. the details of the CAP used in this study is included in discussion and if moved to introduction, clarifies the methods and experimental plans in the study. The importance and applications suggested in the discussion cannot be withdrawn from the results presented in this study are ambiguous and require further studies that shows no negative effects on host cells and tissues.

**Answer:**

**The SMD technology and the respective studies on the topic of mutagenicity against host cells and tissues are now included in the discussion section (page 18, line 389-393).**

5. Although the experiments are performed in an environment similar to human saliva, there are no explanations of the importance of use of this environment and whether other environments may impact on level of antimicrobial efficacy of CAPs.

**Answer:**

**The human saliva was used based on the studies performed by Pratten et al. which showed that usage of saliva is requisite for growth of a robust biofilm and this method was also used in different studies before as follows:**

i) **Pratten J et al.: In vitro studies of the effect of antiseptic-containing mouthwashes on the formation and viability of Streptococcus sanguis biofilms. J Appl Microbiol. 1998;84(6):1149-55;**
ii) **ii) Cieplik F et al.: The impact of absorbed photons on antimicrobial photodynamic efficacy. Front Microbiol. 2015;6:706;**
iii) **iii) Cieplik F et al.: Photodynamic biofilm inactivation by SAPYR--an exclusive singlet oxygen photosensitizer. Free Radic Biol Med. 2013;65:477-87.**
